# Trends in use of neonatal CPAP: a population-based study

**DOI:** 10.1186/1471-2431-11-89

**Published:** 2011-10-17

**Authors:** Christine L Roberts, Tim Badgery-Parker, Charles S Algert, Jennifer R Bowen, Natasha Nassar

**Affiliations:** 1 Clinical and Population Perinatal Health Research, Kolling Institute of Medical Research, University of Sydney, Department of Obstetrics & Gynaecology, Level 2, Building 52, Royal North Shore Hospital, St Leonards, NSW, 2065, Australia; 2 NSW Department of Health, North Sydney, Australia; 3 Department of Neonatology, Royal North Shore Hospital, Sydney, Australia

## Abstract

**Background:**

Continuous positive airway pressure (CPAP) is used widely to provide respiratory support for neonates, and is often the first treatment choice in tertiary centres. Recent trials have demonstrated that CPAP reduces need for intubation and ventilation for infants born at 25-28 weeks gestation, and at > 32weeks, in non-tertiary hospitals, CPAP reduces need for transfer to NICU. The aim of this study was to examine recent population trends in the use of neonatal continuous positive airway pressure.

**Methods:**

We undertook a population-based cohort study of all 696,816 liveborn neonates ≥24 weeks gestation in New South Wales (NSW) Australia, 2001-2008. Data were obtained from linked birth and hospitalizations records, including neonatal transfers. The primary outcome was CPAP without mechanical ventilation (via endotracheal intubation) between birth and discharge from the hospital system. Analyses were stratified by age ≤32 and > 32 weeks gestation.

**Results:**

Neonates receiving any ventilatory support increased from 1,480 (17.9/1000) in 2001 to 2,486 (26.9/1000) in 2008, including 461 (5.6/1000) to 1,465 (15.8/1000) neonates who received CPAP alone. There was a concurrent decrease in mechanical ventilation use from 12.3 to 11.0/1000. The increase in CPAP use was greater among neonates > 32 weeks (from 3.2 to 11.8/1000) compared with neonates ≤32 weeks (from 18.1 to 32.7/1000). The proportion of CPAP > 32 weeks initiated in non-tertiary hospitals increased from 6% to 30%.

**Conclusions:**

The use of neonatal CPAP is increasing, especially > 32 weeks gestation and among non-tertiary hospitals. Recommendations are required regarding which infants should be considered for CPAP, resources necessary for a unit to offer CPAP and monitoring of longer term outcomes.

## Background

Continuous positive airway pressure (CPAP) is used widely to provide respiratory support for neonates, and is often the first treatment choice in tertiary centres [1,2]. There are multiple ways of providing CPAP (eg via underwater bubble CPAP, flow driver or ventilator), different patient interfaces (eg binasal prongs, a single nasopharyngeal prong, face mask, high flow nasal cannula) and various levels of water pressure may be used (usually 4-8 cm water). For extremely preterm neonates, CPAP is an alternative to intubation and mechanical ventilation [3] and at later gestational ages, CPAP may be an alternative to headbox oxygen therapy [4]. CPAP is an attractive option for supporting neonates with respiratory distress, because it preserves spontaneous breathing, does not require endotracheal intubation, and may result in less lung injury than mechanical ventilation [2]. Older and larger neonates appear to be managed effectively using CPAP as the initial and primary method for support without the need for surfactant [1]. However, a significant proportion of neonates born very preterm, particularly prior to 28 weeks, may require combinations of respiratory support modes, which could include surfactant treatment, endotracheal intubation and mechanical ventilation and/or CPAP.

Until recently, there was a lack of data from randomized controlled trials (RCTs) on the effectiveness of CPAP [4,5]. Between 2002 and 2006, Buckmaster and colleagues conducted an RCT comparing CPAP with headbox oxygen for neonates > 31 weeks born in non-tertiary hospitals and found CPAP reduced the need for transfer to a neonatal intensive care unit (NICU) [6]. Trials in very preterm babies published since 2008 suggest that starting CPAP at birth may have important benefits, with 50% of babies 25-28 weeks gestation never requiring intubation and ventilation, and that neonates of this gestational age who commence CPAP from birth have no increased risk of death or bronchopulmonary dysplasia, and in fact are less likely to be on oxygen at 28 days of age [7,8].

Other potential advantages of CPAP compared with intubation and subsequent conventional mechanical ventilation include lower costs, easier operation, potentially fewer risks, and less training [1]. Nevertheless, CPAP is still considered resource-intensive, requiring skilled and experienced staff to ensure the success of the treatment [5] and may result in an increased risk of pneumothorax and nasal trauma [6,8]. The aim of this study was to use population data to examine statewide trends in CPAP use. An additional aim was to assess whether CPAP use changed in hospitals that participated in the Buckmaster CPAP trial [6].

## Methods

### Study population

The study population included all live births in New South Wales (NSW), Australia, from January 2001 to December 2008 with a gestational age of at least 24 weeks and for whom the birth record linked to at least one hospital record. Neonates transferred interstate within 7 days of birth (*N *= 585, < 0.1%) were excluded because no further information was available for these neonates. New South Wales is the most populous state in Australia with a population of 7.2 million and over one-third of all Australian births [9].

### Data sources

The data for this study were obtained from the NSW Midwives Data Collection (MDC) and the NSW Admitted Patient Data Collection (APDC). The MDC (referred to as 'birth' records) is a population-based surveillance system covering all livebirths and stillbirths in NSW. The information is recorded by either the midwife or medical practitioner attending the birth, and includes demographic, medical and obstetric information on the mother and information on the labour, delivery and condition of the neonate. The APDC (referred to as 'hospital' records) is a census of all inpatient admissions (public and private) in NSW. It includes a range of demographic data and clinical information. The diagnoses and procedures related to the admission are coded according to the 10th revision of the International Statistical Classification of Diseases and Related Health Problems, Australian Modification (ICD-10-AM) and the affiliated Australian Classification of Health Interventions, respectively.

The birth record and the infant hospital record associated with the birth were linked for each neonate. Hospital records were also linked longitudinally to identify hospital-to-hospital transfers and readmissions. Probabilistic record linkage was conducted independent of the research by the NSW Centre for Health Record Linkage [10]. No health information is used for linkage, and at no time is identifying information made available to researchers. The study was approved by the NSW Population and Health Services Research Ethics Committee.

### Outcome and explanatory factors

The primary outcome was whether a neonate received CPAP or mechanical ventilation (via endotracheal intubation) between birth and initial discharge from the hospital system, identified from any of 20 procedure fields in any of the neonate's hospital records. A NSW validation study found that any mechanical ventilation and CPAP are reliably reported in population health data [11]. For analysis, neonates were classified as 'ventilation' if they received mechanical ventilation, whether or not they received CPAP, and as 'CPAP' if they received CPAP alone and did not receive mechanical ventilation in the period from birth to discharge from the hospital system. Neonatal transfer to a NICU was a secondary outcome.

Hospitals were categorised as 'tertiary', 'CPAP trial' and 'other non-tertiary'. Tertiary hospitals (n = 7) have a Level III neonatal intensive care unit (NICU) which provides mechanical ventilation and care for neonates with severe and/or complex illness [12]. The CPAP trial hospitals were the five NSW hospitals that participated in the Buckmaster CPAP trial [6]. Participation in this trial required a paediatric registrar onsite 24-hours and nursing staff trained in use of CPAP for neonates. The 'other non-tertiary' hospitals (n = 78) included all other public and private hospitals at which babies were born in NSW, and have service levels ranging from general practitioner or midwife care with low level neonatal care to special care nurseries. The neonatal factors available for analysis included gender, plurality (singleton vs multiple), gestational age, small for gestational age (SGA, < 10th percentile) and large for gestational age (LGA, > 90th percentile), [13] Apgar score less than 4 at 1 minute and Apgar score less than 7 at 5 minutes. Gestational age is reported in completed weeks of gestation as determined by the best clinical estimate, including early ultrasound (> 97%) and date of the last menstrual period. Only factors that are accurately reported were included in the analyses [14-18].

### Analysis

We determined rates of CPAP over time and by neonate characteristics, gestational age and hospital of CPAP initiation. Gestational age was categorised as ≤32 weeks and > 32 weeks, based on national policy recommending neonates ≤32 weeks be delivered in tertiary centres [19].

## Results

The study included 696,816 live births of at least 24 weeks gestation born in NSW between 2001 and 2008, increasing from 82,542 in 2001 to 92,461 in 2008. Of all infants, 191,511 (27.5%) were born at tertiary hospitals, 94,390 (13.5%) at CPAP-trial hospitals and 410,915 (59.0%) at other non-tertiary hospitals. Overall 6,188 (8.9/1000 livebirths) neonates received CPAP alone and 8,229 (11.8/1000) received mechanical ventilation with or without CPAP, including 3,700 (5.3/1000) who received both mechanical ventilation and CPAP. Compared to neonates who did not receive any ventilatory support, those receiving CPAP alone were more likely to be male (59% versus 51%), of multi-fetal pregnancies (21% versus 3%), born at < 37 weeks (69% versus 5%) and have low Apgar scores at 1 (10% versus 2%) and/or 5 minutes (9% versus 1%) (Table 1). Twins and triplets were 8.6 times more likely than singletons to receive CPAP alone, but after adjusting for gestational age this risk decreased substantially (adjusted RR: 1.13; 95% CI: 1.07-1.20). For the gestational ages 31 through 36 weeks, more neonates received CPAP alone than were ventilated. Ventilation numbers only exceeded CPAP alone at ≤30 weeks gestation (Table 1). The gestation-specific rates of CPAP alone were 261.9/1000 among neonates ≤32 weeks, 51.3/1000 at 33-36 weeks and 2.9/1000 at ≥37 weeks. Among infants who required respiratory support, those with transient tachypnea were more than twice as likely to be managed with CPAP alone (RR = 2.5; 95% CI 2.3-2.7) compared to those with other diagnoses; 65% of infants with transient tachypnea as one of their admitting diagnoses and requiring respiratory support received CPAP alone.

**Table 1:** Characteristics of neonates receiving CPAP or mechanical ventilation, NSW 2001-2008

Infant and hospital characteristics	CPAP alone		Mechanical ventilation*		No ventilation or CPAP	
	N = 6188		N = 8229		N = 682,399	
	n	(%)	n	(%)	n	(%)
Male	3625	(58.6)	4908	(59.6)	350346	(51.3)
Female	2563	(41.4)	3321	(40.4)	332053	(48.7)
						
Singleton	4870	(78.7)	6846	(83.2)	663798	(97.3)
Multiple	1318	(21.3)	1383	(16.8)	18601	(2.7)
						
Gestational age						
24-26	80	(1.3)	1008	(12.3)	207	(0.03)
27-29	559	(9.0)	1506	(18.3)	276	(0.04)
30-32	1734	(28.0)	1355	(16.5)	2337	(0.3)
33-34	1115	(18.0)	803	(9.8)	7598	(1.1)
35-36	800	(12.9)	682	(8.3)	26332	(3.9)
37-38	681	(11.0)	995	(12.1)	151713	(22.2)
39-40	803	(13.0)	1282	(15.6)	371034	(54.4)
41-42	413	(6.7)	597	(7.3)	122056	(17.9)
> 42	3	(0.05)	1	(0.01)	846	(0.12)
						
Size at birth						
< 10th centile	636	(10.3)	1127	(13.7)	64996	(9.5)
10th-90th centile	4865	(78.6)	6241	(75.8)	545275	(79.9)
> 90th centile	687	(11.1)	861	(10.5)	72128	(10.6)
						
Apgar at 1 minute						
< 4	626	(10.2)	2450	(30.1)	10011	(1.5)
≥4	5538	(89.8)	5701	(69.9)	670734	(98.5)
						
Apgar at 5 minutes						
< 7	529	(8.6)	2375	(29.1)	6355	(0.9)
≥7	5634	(91.4)	5783	(70.9)	674538	(99.1)

* with or without CPAP

**Figure 1:**
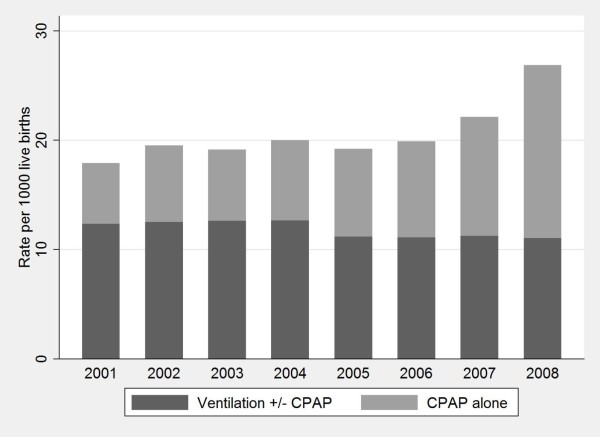
**Trend in ventilation and CPAP rates among neonates, NSW 2001-2008**.

**Figure 2:**
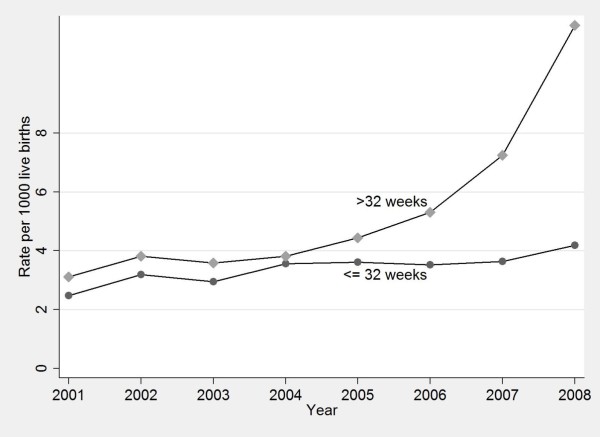
**Trend in the CPAP rate per 1000 live births, by gestational age, NSW 2001-2008**.

The number and rate of neonates receiving any ventilatory support increased from 1,480 (17.9/1000) in 2001 to 2,486 (26.9/1000) in 2008 (P < 0.001), including an increase from 461 (5.6/1000) to 1,465 (15.8/1000) neonates who received CPAP alone (P < 0.001) (Figure 1). There was a concurrent decrease in any use of mechanical ventilation from 12.3 to 11.0/1000 livebirths. However, there was no significant change in the use of combined mechanical ventilation and CPAP (P = 0.52). The relative increase in the use of CPAP alone was greater among neonates > 32 weeks (increasing 3.7-fold from 3.2/1000 in 2001 to 11.8/1000 livebirths in 2008) compared with neonates aged ≤32 weeks (increasing 1.8-fold from 18.1 to 32.7/1000 livebirths) (Figure 2).

**Figure 3:**
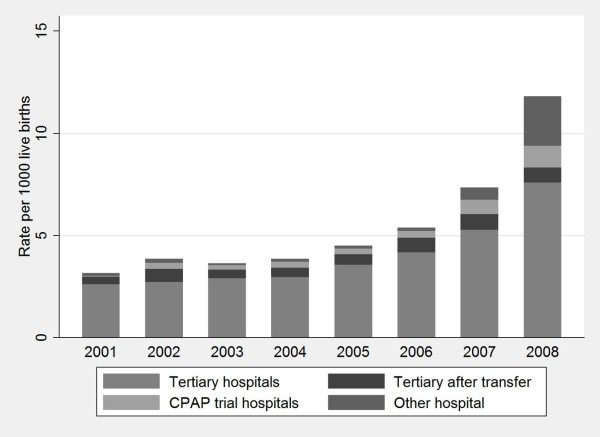
**CPAP initiation for neonates > 32 weeks by type of hospital**.

When examined by hospital of initiation, 89% of CPAP was initiated in tertiary centres, including 8% that was initiated following neonatal transfer. Although in absolute numbers CPAP initiated in tertiary centres among neonates > 32 weeks increased, the proportion provided by tertiary hospitals declined from 94% (3.0/1000) in 2001 to 70% (8.3/1000) in 2008 (P < 0.001, Figure 3). Compared with 2003-2006 (the period of Buckmaster trial), the number of neonates > 32 weeks receiving CPAP alone at the CPAP trial hospitals doubled in 2007. From 2001 to 2006, between 3 (2.5%) and 11 (9.6%) other non-tertiary hospitals provided CPAP to neonates (Figure 3). In 2007, this number had doubled, with 22 (23.4%) hospitals providing CPAP to 56 neonates, and by 2008 further increased to 27 hospitals (28.1%) treating 222 infants. From 2001 to 2008, the proportion of neonates born outside a tertiary centre and transferred to a NICU declined slightly from 755 (1.2%) to 745 (1.1% P < 0.001). Neonates who received CPAP at the CPAP trial hospitals were significantly less likely to be transferred to a NICU than neonates who received CPAP at other non-tertiary hospitals (13.6% versus 20.1%, P = 0.03).

## Discussion

Use of CPAP without mechanical ventilation for neonates increased from 2001 to 2008, with a particularly notable rise among infants of > 32 weeks gestation and at non-tertiary hospitals in 2008. Although the rates of CPAP use were highest among infants ≤30 weeks, in terms of absolute numbers of neonates exposed, the burden was higher among more mature neonates. The relatively slower increase in CPAP use among neonates ≤32 weeks may relate to greater difficulty in supporting very premature neonates on CPAP alone without a period of mechanical ventilation or may reflect the lack of evidence regarding use of CPAP in very preterm neonates available during the study period. Trials in very preterm neonates published from 2008 support the consideration of CPAP as an alternative to intubation and surfactant, [7,8] and as these findings are translated into practice we may see a further increase in CPAP rates.

CPAP use among neonates > 32 weeks increased slowly from 2001 through 2004, followed by greater increases, particularly in 2008. During this period, there was no increase in the temporal trend of births ≤32 or > 32 weeks gestation. The overall increase in neonates receiving CPAP was offset by a small decrease in rate of mechanical ventilation, resulting in a significant increase in total number of infants receiving ventilatory support (CPAP or mechanical ventilation). This suggests that the increase was primarily due to an increase in CPAP in neonates who would previously have received only supplemental oxygen in either a tertiary or non-tertiary unit. The 2007 increase coincided with publication of the Buckmaster CPAP trial results, which showed CPAP for selected neonates at appropriately resourced non-tertiary hospitals could reduce transfers to a NICU [6]. At the hospitals involved in the CPAP trial the number of neonates receiving CPAP doubled after the trial, implying that neonates who would previously have been randomized to oxygen were instead being given CPAP.

The use of large, linked, validated population-based databases that provide information on all neonates is a strength of this study. However, these data do not have detailed clinical information such as the severity of disease, use of surfactant or the duration of CPAP. Furthermore, the temporal sequence of events (eg CPAP, mechanical ventilation, pneumothorax) cannot be determined, only that the events occurred during an admission. This limits the ability to assess complications or reasons for changes in the respiratory support methods.

Nasal CPAP has been adopted by many NICUs as a way of reducing rates of bronchopulmonary dysplasia in premature neonates, but assessment of its benefits is complicated by questions about the simultaneous effects of concomitant surfactant treatments and other NICU interventions [20]. Most research into the potential benefit of CPAP has used a study population of very preterm or extremely preterm neonates who were delivered in tertiary referral hospitals. Little is known about the benefits of CPAP use in more mature neonates in tertiary NICUs. The Buckmaster trial compared CPAP use with supplemental oxygen in neonates > 30 weeks gestation in non-tertiary centres [6] to prevent transfer of neonates for intensive care. The study showed a reduction in both treatment 'failure' (RR = 0.54; 95% CI 0.32, 0.91) and the rate of up-transfer (RR = 0.51; 95% CI 0.31, 0.89), but did not show any statistically significant reduction in outcomes such as length of admission. The results also show an increased risk of pneumothorax in the CPAP arm but the confidence interval is wide (RR = 2.76; 95% CI 1.02, 7.48). The possibility of increased rates of pneumothorax has been a concern with use of CPAP, and the COIN trial [8] reported a rate of pneumothorax three times higher in the CPAP group compared with the mechanical ventilation group. However, the recently published results of the SUPPORT trial found no difference in the rates of pneumothorax for extremely preterm neonates randomized to initial treatment with either CPAP or endotracheal administration of surfactant [7]. Further, the long term consequences of CPAP remain undetermined and need to be monitored.

Although our findings highlight that most neonates treated with CPAP are cared for in tertiary centres, there was an increase in the proportion treated outside these hospitals. Our study found that most non-tertiary non-CPAP-trial hospitals that provided CPAP support treated relatively few neonates in 2007-2008; and this may be inadequate to ensure safety and cost-effectiveness of the intervention. CPAP is resource-intensive and caution has been advised with the use of CPAP in units that are not well staffed or experienced in its use [21-23]. Furthermore, it is important that the availability of CPAP facilities does not lead to complacency regarding policies of antenatal transfer of high risk pregnancies, particularly as in utero transfer has been demonstrated to be more beneficial and improve neonate outcomes [24].

Buckmaster estimated that on average, across an neonatal population, a cost saving of ~AU$1,700 would accrue for every neonate treated with CPAP [6]. However, increased use of CPAP is likely to increase costs at an individual hospital if additional resources, such as experienced staff and ongoing monitoring of CPAP neonates, are required [2]. Additional costs associated with CPAP use in non-tertiary hospitals may be offset by a reduction in neonatal transfers, decreased length of stay or better outcomes for neonates [24] and should be investigated. Although the Buckmaster CPAP trial did show a benefit in reduced transfers,[6] it is not known if this remains true in the wider group of hospitals now providing CPAP and among the potentially broader group of neonates exposed, especially given the small numbers treated at each hospital. Recommendations should be developed regarding which neonates should be considered for CPAP, and the appropriate resources necessary for a unit to offer CPAP. In addition, longer term outcomes for neonates who receive CPAP need to be monitored.

## Conclusions

CPAP use has increased since 2001, most notably among neonates > 32 weeks and this represents an extension of ventilatory support rather than replacement of mechanical ventilation. Furthermore, CPAP use appears to have increased in non-tertiary hospitals following publication of a randomized trial showing CPAP decreased the need for neonatal transfer. Our study highlights the need for recommendations about which neonates should be considered for CPAP and the appropriate resources necessary to offer CPAP.

## Abbreviations

ACHI: Australian Classification of Health Interventions; APDC: NSW Admitted Patients Data Collection; CHeReL: Centre for Health Record Linkage; CPAP: continuous positive airway pressure; MDC: NSW Midwives Data Collection; NSW: New South Wales; SGA: Small for gestational age; LGA: Large for gestational age

## Competing interests

The authors declare that they have no competing interests.

## Authors' contributions

Christine Roberts, Charles Algert and Natasha Nassar developed the concept and design of the study and were responsible for overall drafting of the manuscript. Tim Badgery-Parker conducted the analysis and drafted the methods and results. Jennifer Bowen revised the manuscript for important intellectual content. All authors contributed to the interpretation of data and had final approval of the manuscript to be published.

## Pre-publication history

The pre-publication history for this paper can be accessed here:

http://www.biomedcentral.com/1471-2431/11/89/prepub
